# Rapid identification of fungi in culture-negative clinical blood and respiratory samples by DNA sequence analyses

**DOI:** 10.1186/s13104-016-2097-0

**Published:** 2016-06-07

**Authors:** Farida Sidiq, Matt Hoostal, Scott O. Rogers

**Affiliations:** Department of Biological Sciences, Bowling Green State University, Bowling Green, OH 43403 USA

**Keywords:** Molecular diagnostics, Mycosis, *Candida*, *Aspergillus*

## Abstract

**Background:**

Clinical diagnoses of fungal infections often rely upon culture techniques followed by microscopic examination of positive cultures and histopathological specimens. Culturing of microorganisms is prone to false negatives, while microscopy methods can be complicated by atypical phenotypes and organisms that are morphologically indistinguishable in tissues. Delays in diagnoses (or the lack thereof) and inaccurate identification of infectious organisms contribute to increased morbidity and mortality in patients.

**Methods:**

Two-hundred randomized, heterogeneous patient blood and respiratory samples that were culture-negative were tested using polymerase chain reaction (PCR) amplification of internal transcribed spacer regions of ribosomal RNA genes utilizing panfungal primers. Amplicons were sequenced, subjected to sequence similarity searches, and compared using phylogenetic analyses.

**Results:**

Thirteen fungal sequences were detected in three whole-blood samples and nine respiratory samples. Bioinformatic analyses were performed which indicated the presence of multiple pathogens and potential pathogens.

**Conclusions:**

The results from this pilot study demonstrate the utility of PCR assays and sequence analyses in clinical tests for fungi to facilitate rapid diagnosis and appropriate treatments to deal with the false negatives from culture results.

## Background

Invasive aspergillosis (IA) and invasive candidiasis (IC) are two of the most frequently encountered opportunistic fungal diseases in immunocompromised individuals and are also two of the most frequent nosocomial fungal infections [1–8]. In addition, the emergence of normally non-pathogenic fungi as causative agents of disease has become a serious concern with increases of transplant surgeries [7], immunosuppressive therapies [9], individuals with HIV/AIDS [10, 11], and patients with underlying primary diseases such as hematological malignancies and cancer [12]. Multiple-cause-of-death records from the period 1980–1997 reported that multiple-cause mortality due to invasive mycoses increased over 300 % and that mortality from IA increased over 350 % [13]. Furthermore, non-*Aspergillus* fungal infections in organ transplant patients have increased and resulted in increases of morbidity and mortality [14].

Increases in opportunistic fungal infections and immunocompromised populations have led to the need to accurately identify fungal pathogens rapidly and specifically. Patient morbidity and mortality statistics show that delays in diagnosis and appropriate treatment contribute significantly to poor prognoses [15]. Consensus from the international community for diagnostic criteria for proven, probable, and possible invasive fungal infections (IFI) has been recalcitrant [5, 16]. Traditionally, IFI have been characterized from phenotypic identification based on positive cultures or from histology of body sites [17].

Several problems exist with these approaches. First, patients with suspected infections may not be healthy enough for invasive biopsy procedures due to immunosuppressive therapies or other conditions [9, 11]. Physical trauma to skin from biopsies, and subsequent exposure to nosocomial pathogens presents inherent risks for additional infections. In addition, misidentification of fungi, or failure to identify any causative microorganisms, in histological and cytological samples [18, 19] present critical challenges to prompt and appropriate treatment. Non-invasive procedures such as computed tomography (CTs) or X-rays may fail to reveal mycetomas and nephrotic lesions promptly enough for effective treatment. In addition, high proportions of false-negative results in clinical tests are possible, with reported sensitivities of 8.3 [20] to 31 % [21]. Emerging variants of pathogenic and assumed non-pathogenic fungi may be overlooked. Cultures may also exhibit atypical morphology [22, 23], multiple morphologies, or require lengthy incubations of several weeks or more for colony growth [24], as well as expert interpretations. Finally, the presence of more than one species in a sample may contribute to a culture-negative interpretation [25].

### Aim of this study

The present study retrospectively tested randomly chosen blood and respiratory samples that were reported to be culture-negative for bacterial and fungal pathogens. The specific objectives were to amplify ribosomal DNA (rDNA) internal transcribed spacer (ITS) regions by polymerase chain reaction (PCR) using panfungal ITS primers, sequence the major amplicons, compare PCR-positive samples to patient information (such as age and gender) to identify associations between potential fungal pathogens and subsets of heterogeneous, randomized patient populations, and finally to perform phylogenetic analysis to infer taxonomic classifications for each of the fungal sequences found. ITS regions have been used to delineate fungi to the species level, and often the strain level [23, 26, 27]. A relatively innovative characteristic of this research is that rather than targeting specific patients based upon known patient demographics or other risk factors associated with increased incidence of fungal infection [28, 29], molecular testing for fungal infections was performed on randomly chosen patients. The primary aim of this study was to determine whether the utilization of standard molecular techniques (specifically PCR amplification and sequencing of rDNA ITS regions) could provide essential improvements to laboratory identification of uncommon and emerging fungal pathogens.

## Methods

### Sample collection and transportation

Samples were collected aseptically by hospital personnel at the University of Michigan Hospital Clinical Microbiology and Virology Laboratories (Ann Arbor, MI). After clinical testing (see below) using the BacT/Alert blood culture instrument (bioMerieux, Inc.) and standard microbiological culture techniques, samples of 100 culture-negative blood (for bacteria and fungi) and 100 culture-negative (for bacteria) respiratory samples were collected for study that were obtained from randomized patients in heterogeneous populations in the hospital within a 1-year period in 2005 and 2006. Only one sample was obtained from each patient. Approval was obtained from the Human Subjects Institutional Review Boards at the University of Michigan and Bowling Green State University. The samples were deidentified and obtained from discarded microbiology laboratory specimens. Because of this, the study was exempt from informed consent procedures. The samples were immediately transported on ice to the Bowling Green State University laboratory and stored at −20 °C. Pre-sterilized single-use needles were used for blood preparations and pre-sterilized aerosol-resistant pipet tips were used for preparation of respiratory samples. Aliquots were stored at −20 °C prior to testing.

### Sample and patient information

Thirty-seven of the whole-blood samples had been cultured anaerobically, and 63 had been cultured aerobically using BacT/Alert FAN (bioMerieux, Inc.) media by University of Michigan Hospital personnel. Collection volumes ranged between 1 (pediatric) and 10 ml (adult) and cultures were held for an average of 5 days before they were considered culture-negative for fungal and bacterial pathogens. Respiratory samples consisted of bronchoalveolar lavage (BAL, n = 25), sinus (n = 1) and sputum (n = 74) specimens and had been cultured aerobically by University of Michigan Hospital personnel. Volumes collected were between 2 and 10 ml and cultures were considered negative after 2 days if no growth was observed.

Patient age ranged from less than 1-year old to 91 years old. Fifty-nine patients were between the ages of <1–20 years old (29.5 %), thirty were 21–40 years old (15 %), 62 were 42–60 years-old (31 %), 42 were 61–80 years old (21 %), and seven were 81–91 years-old (3.5 %). One-hundred and three patients were male (51.5 %) and 97 patients were female (48.5 %). Most samples (n = 137) were collected from non-Intensive Care Units (68.5 %), whereas 63 samples were collected from Intensive Care Units (ICU) (31.5 %).

### DNA extraction

A cetyltrimethylammonium bromide (CTAB) DNA extraction method [30–33] with slight modifications was performed on the samples. This method eliminates biomolecules and other chemicals that often inhibit PCR (and other) reactions through the utilization of differential precipitation steps. Positive controls consisting of blood or respiratory samples to which had been added either *Aspergillus flavus* or *Aspergillus fumigatus* ATCC^®^ MYA-4609™ cells were performed at the same time. Additionally, negative controls were included that consisted of 200 μl of sterilized reverse osmosis water (18.2 MΩ, <1 ppb TOC).

### PCR, cloning, and DNA sequencing

**Fig. 1:**
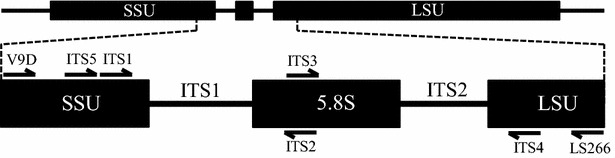
Locations for the panfungal primers in the rDNA internal transcribed spacer (ITS) region. *Arrows* indicate locations and orientations of primers used in this study. SSU and LSU represent the small and large rRNA gene subunits, respectively. Primer sequences are provided in Table 1

Primer combinations (Table 1; Fig. 1) targeting the internal transcribed spacer 1 (ITS1) region (primer pair ITS2/ITS5 [34]), the ITS2 region (primer pairs ITS3/ITS4Z or ITS4FS [35, 36]), or both regions (primer pairs ITS4Z or ITS4FS/ITS5, LS266/V9D) were used to amplify PCR products utilizing a Bio-Rad PTC-100 Peltier Thermal Cycler (Integrated DNA Technologies, Coralville, IA). Reactions consisted of approximately 1–10 ng genomic DNA, 20 mM (NH_4_)_2_SO_4_, 50 pmol each primer, 1.5 mM MgCl_2_, 200 μM each dNTP, and two units native *Taq* DNA polymerase (Fermentas, Glen Burnie, MD). The PCR program [23, 37] consisted of the following steps: 95 °C for 1 min, followed by 35 cycles of 94 °C for 1 min, annealing temperature (50, 52 or 55 °C) for 4 min, and 72 °C for 4 min; and then a final extension of 72 °C for 10 min. PCR products that exhibited robust bands on 1 % agarose (in TBE @ 5 V/cm for 1 h) were purified using QIAquick PCR purification kits (QIAGEN, Valencia, CA), as per the manufacturer’s protocol. Purified eluates were confirmed on 1 % agarose gels, and were stored at −20 °C.

**Table 1:** Panfungal internal transcribed spacer (ITS) primers utilized in this study

Primer name	Sequence (5′–3′)	Reference
ITS2	GCTGCGTTCTTCATCGATGC	20
ITS3	GCATCGATGAAGAACGCAGC	20
ITS4FS	TCCTCCGCTTATTNATATGC^a^	F. Sidiq, unpublished
ITS4Z	TCCTCCGCTTATTRATATGC^a^	G. Zhang, unpublished
ITS5	GGAAGTAAAAGTCGTAACAAGG	20
LS266	GCATTCCCAAACAACTCGACTC	21
V9D	TTAAGTCCCTGCCCTTTGTA	22

^a^N = A, C, G, or T and R = A or G

When PCR products exhibited multiple robust bands on 1 % agarose gels, each band was excised and then purified using a QIAquick Gel Extraction kit, as per manufacturer’s protocol. These were cloned into pCR2.1-TOPO vectors (TOPO TA Cloning kit for sequencing, Invitrogen, Carlsbad, CA), as per manufacturer’s protocol. For transformations, 4 μl of cloning products were added to a vial of One Shot™ Chemically Competent *E. coli* cells (Thermo Fisher Scientific, Carlsbad, CA), as per manufacturer’s protocol, and were spread on Luria–Bertani Difco™ LB Agar plates (Becton, Dickson and Company, Sparks, MD) with 50 μg/ml ampicillin (Sigma-Aldrich, St. Louis, MO) supplemented with 40 μl 5-bromo-4-chloro-3-indoxyl-beta-d-galactopyranoside (X-gal, 20 mg/ml, Gold Biotechnology, St. Louis, MO). Duplicate plates were prepared for each sample using 50 or 75 μl of transformed cells and were then incubated overnight at 37 °C.

At least ten recombinant colonies were selected from each plate and subcultured on Luria–Bertani agar (Difco™ LB Agar) supplemented with 50 μg/ml ampicillin. After overnight incubation, 5–10 colonies were selected from each cloning reaction and inoculated into 4 ml Difco™ LB broth with 50 μg/ml ampicillin and incubated overnight at 37 °C with shaking. Plasmids were isolated using the Cyclo-Prep miniprep plasmid DNA purification kit (Amresco, Solon, OH).

Plasmids were analyzed for inserts by digestion with *Eco*RI or by PCR amplification reactions containing the following components: 1× *Taq* buffer with (NH_4_)_2_SO_4_ [750 mM Tris–HCl (pH 8.8) 200 mM (NH_4_)_2_SO_4_, 1 % Tween 20], 200 μM each dNTP, 1.25 units native *Taq* DNA polymerase (Fermentas, Inc., Glen Burnie, MD), 1.5 mM MgCl_2_ and 50 pmol of M13 primers [M13F(−20): GTAAAACGACGGCCAG, M13 reverse: CAGGAAACAGCTATGAC]. PCR was performed using the following program: 95 °C for 4 min, 30 cycles of 95 °C for 1 min, 45 °C for 2 min, 72 °C for 2 min, and a final extension at 72 °C for 10 min. All purified PCR and plasmid isolations were commercially sequenced by Geneway, LLC (Hayward, CA, USA) using Sanger sequencing methods.

### Bioinformatic analyses of sequences

The resulting ITS sequences were compared to GenBank entries in the National Center for Biotechnology Information (NCBI) database. BLASTn was used with default parameters and sequences with at least 96 % maximum identity were aligned with sequences from the blood and respiratory samples using MAFFT version 5.7 [38] from the bioinformatics toolkit at the Max Planck Institute for Developmental Biology (http://www.toolkit.tuebingen.mpg.de/sections/alignment). Reference sequences were collected if they were from published studies and had been identified to at least the species level. Multiple sequence alignments were visually inspected and curated for subsequent analyses. Phylogenetic analysis was performed with MEGA v. 6 [39] using maximum likelihood and neighbor-joining, with a maximum composite likelihood model. Since phylogenetic reconstructions from each method have differing underlying assumptions, congruent trees derived from the two methods increased confidence in tree topology [40]. Bootstrap analysis (1000 replications) was also performed as a measure of support for tree nodes [41]. The best-fit nucleotide substitution model, based on the corrected Akaike (AICc) and Bayesian Information Criteria (BIC), was determined. For sequences corresponding to the ITS1 region, hierarchical likelihood ratio tests ascertained that the Kimura 2-Parameter substitution model with invariant rate differences among sites was the optimal evolutionary model for phylogenetic inference. For sequences corresponding to the ITS2 region, as well as both regions, the Jukes-Cantor substitution model with gamma-distributed rate differences among sites was determined as the best-fit evolutionary model for phylogenetic reconstruction.

### Statistical analyses

Statistical analyses were conducted with Minitab v. 17. Unpaired t tests were used to compare means of patient length of stay (LOS) or age for PCR-positive samples to PCR-negative samples. Additionally, Fisher’s exact test with a 2 × 2 contingency table was used for categorical data to compare the frequency of patients in ICU for PCR-positive patients and PCR-negative patients, as well as the frequency of male and female patients.

## Results

**Fig. 2:**
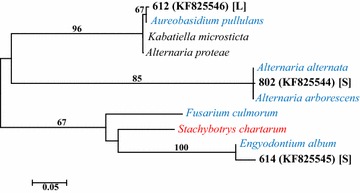
Maximum likelihood phylogenetic tree derived from alignments of ITS1 sequences. The best-fit nucleotide substitution model for the sequence dataset, based on the corrected Akaike (AICc) and Bayesian Information Criteria (BIC), was determined using MEGA v. 6 [39]. Hierarchical likelihood ratio tests ascertained that the Kimura 2-Parameter substitution model with invariant rate differences among sites was the optimal evolutionary model for phylogenetic inference. Fungal sequences collected from samples are in *bold black font*, labeled with the patient ID followed by NCBI (GenBank) accession numbers in *parentheses*. Genus and species are provided for the reference sequences. *Red font* indicates a known human pathogen, and *blue font* indicates species that have been documented as opportunistic pathogens in immunocompromised patients. Bootstrap values (1000 replications) are provided to indicate support levels for tree nodes. Unlabeled branches have bootstrap values below 50 %. Specimen type: [*L*] = *BAL* bronchoalveolar lavage, [*S*] sputum

**Fig. 3:**
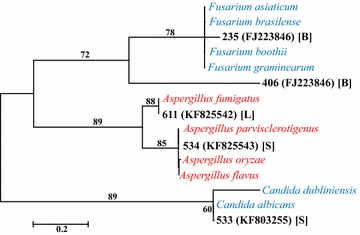
Maximum likelihood phylogenetic tree derived from alignments of ITS2 sequences. The best-fit nucleotide substitution model for the sequence dataset, based on the corrected Akaike (AICc) and Bayesian Information Criteria (BIC) was determined using MEGA v. 6 [39]. Hierarchical likelihood ratio tests ascertained that the Jukes-Cantor substitution model with gamma-distributed rate differences among sites was the optimal evolutionary model for phylogenetic inference. Fungal sequences collected from samples and reference sequences are labeled as in Fig. 2. Bootstrap support values are indicated as in Fig. 2. Specimen type: [*B*] blood, [*L*] = *BAL* bronchoalveolar lavage, [*S*] sputum

**Fig. 4:**
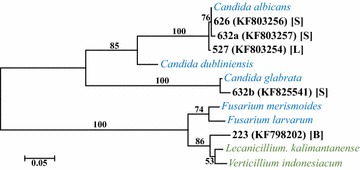
Maximum likelihood phylogenetic tree derived from alignments of ITS1 and ITS2. The best-fit nucleotide substitution model for the sequence dataset, based on the corrected Akaike (AICc) and Bayesian Information Criteria (BIC). Hierarchical likelihood ratio tests ascertained that the Jukes-Cantor substitution model with gamma-distributed rate differences among sites was the optimal evolutionary model for phylogenetic inference. Fungal sequences collected from samples and reference sequences are labeled as in Fig. 2. *Green font* indicates species that have been isolated from arthropod pathogens, but have occasionally been associated with human infections in immunocompromised patients [42]. Bootstrap support values are indicated as in Fig. 2. Specimen type: [*B*] = blood; [*L*] = *BAL* bronchoalveolar lavage, [*S*] sputum

Fungal DNA was detected in nine of the 100 respiratory culture-negative samples and three of the 100 whole-blood culture-negative samples (Table 2). Sample type, patient sex, age, location in the hospital, putative fungal type, maximum identity, and sequence lengths compared are summarized in Table 2. Seven of the twelve samples that were positive for fungal DNA were from patients in an ICU. However, none of the variables, including presence in the ICU, patient LOS, sex, or age differed significantly with respect to the presence or absence of fungal DNA (Fisher’s exact p values = 0.054–0.136). A combination of BLASTn and phylogenetic reconstructions provided indications of the taxa closest to those determined from the patient samples. Maximum likelihood and neighbor-joining methods generally confirmed the taxonomic relationships inferred by the BLASTn results (Figs. 2, 3, 4). In most instances, high bootstrap values (>80) provided confidence for nodes that clustered observed sequences with reference sequences. The taxa closest to the sequences in blood specimens included *Lecanicillium kalimantanense*, *Cladosporium cladosporioides,* and *Fusarium graminearum*. The taxa closest to the sequences from respiratory samples included four examples of *Candida albicans*, *Aspergillus flavus*, *Aspergillus fumigatus*, *Engyodontium album*, *Candida glabrata*, *Alternaria alternata* and *Aureobasidium pullulans*.

**Table 2:** Summary of patient characteristics and taxonomic affinities of fungi in the samples based on ITS1 and ITS2 sequences

Patient ID (Accession #)	Patient characteristics			Sample source	Putative taxa	Identity (%)/# bp
	Sex	Age	Location			
223 (KF798202)	Female	57	Hematology/oncology	Blood	*Lecanicillium kalimantanense* ^a^	96/827
235 (FJ223846)	Female	62	Dermatology	Blood	*Fusarium graminearum* ^b^	100/217
406 (FJ223846)	Female	<1	Neonatal ICU	Blood	*Cladosporium cladosporioides* ^b^	100/215
527 (KF803254)	Male	44	Medical ICU	BAL	*Candida albicans* ^b^	99/509
533 (KF803255)	Male	21	Pulmonary	Sputum	*Candida albicans* ^b^	99/444
534 (KF825543)	Male	68	Medical ICU	Sputum	*Aspergillus flavus* ^c^	100/236
611 (KF825542)	Female	52	Medical ICU	BAL	*Aspergillus fumigatus* ^c^	100/232
612 (KF825546)	Female	77	Medical ICU	BAL	*Aureobasidium pullulans* ^b ^	99/274
614 (KF825545)	Female	54	Cardiology ICU	Sputum	*Engyodontium album* ^b^	98/201
626 (KF803256)	Female	75	Cardiology	Sputum	*Candida albicans* ^b^	100/560
632a^d^ (KF803257)	Female	73	Pulmonary	Sputum	*Candida albicans* ^b^	100/562
632b^d^ (KF825541)	Female	73	Pulmonary	Sputum	*Candida glabrata* ^b^	98/860
802 (KF825544)	Female	20	Medical ICU	Sputum	*Alternaria alternata* ^b^	96/426

*BAL* bronchoalveolar lavage

^a^Arthropod-associated; Occasional infections in immunocompromised patients [42]

^b^Documented infections of immunocompromised patients

^c^Human pathogen

^d^Two separate prominent sequences were recovered from patient 632 (632a and 632b)

## Discussion

Increases in fungal infections, including increasing diversity in the species infecting immunocompromised patients, as well as difficulties in rapidly diagnosing fungal infections, requires that additional vigilance and more sensitive methods be broadly adopted in order to affect rapid and accurate diagnoses and targeted treatments. This is especially important in cases where the fungi are able to disseminate into multiple tissues where effective therapies become more difficult and dangerous. Fungal infections can initially appear similar to bacterial infections. However, antibiotic treatment is contraindicated for fungal infections because it may further weaken a patient, or exacerbate the fungal infection, by causing the reduction in the number of beneficial bacterial species. Molecular methods are effective in rapid detection of fungal infections. They can be valuable additions to the diagnostic tools available to assess the causative organisms in diseased individuals.

The molecular biology methods used here allowed the detection of sequences from pathogenic and potentially pathogenic fungi in 6.5 % (13 of 200) of the culture-negative specimens. Furthermore, the sequence analyses were sufficient to allow the determination of fungi to at least the level of genus, with identity values of 96–100 % over lengths of 201–850 bp (Table 2). The rDNA ITS regions have been successfully used in taxonomic and phylogenetic studies to circumscribe taxa at the levels of genus and species, and in some cases to the variety/isolate/strain levels [e.g., 23, 26, 27]. Most of the species identified are clinically relevant, while a few are unknown to produce symptoms in humans, or might be the causes of unrecognized symptoms. For example, species of *Aspergillus* and *Aureobasidium* are known human pathogens. All of the specimens that were positive for species in these two genera exhibited 99–100 % identity over 232–274 bp, and thus are likely to be the species indicated (i.e., *Aspergillus flavus, A. fumigatus*, and *Aureobasidium pullulans*). A large number of the fungus-positive specimens exhibited sequences closest to species that have been reported in immunocompromised patients. These were: specimen 235 (100 % over 217 bp to *Fusarium graminearum*); specimen 406 (100 % over 215 bp to *Cladosporium cladosporioides*); specimens 527, 533, 626, and 632a (99–100 % identities over 236–562 bp to *Candida albicans*); specimen 632b (98 % over 860 bp to *Candida glabrata*); and specimen 802 (96 % sequence identity of 426 bp to *Alternaria alternata*). While 98–100 % sequence identities likely indicate that the species designations accurately identify the fungus in the sample, sequence identities ≤97 % are indications that the sequences represent closely related fungal species, possibly within the same genus, but not necessarily the fungus whose sequence matched on the NCBI (GenBank) sequence database. This is the case for the sequence from specimen 802, which most closely matched *Alternaria alternata*, as well as the sequence from specimen 223, which was closest to the *Lecanicillium kalimantanense* sequence from the sequence database. The latter species is most often found on arthropods, but has been reported as a possible human pathogen [42]. It is likely that the sequence from specimen 223 is from a related species whose sequence is not yet represented in the database. Whether this is a common species, whether it has been described scientifically, or whether it is a human pathogen, are unknown.

The greater percentage of PCR-positive results from ICU patient samples compared to total patients concurs with reports that identify increased instances of fungal infections in ICUs [43]. In contrast, the greater levels of PCR-positive female patients relative to females among the total population of subjects studied is contrary to previous studies [44]. However, none of the associations with any of the variables, including presence in the ICU, sex, age, or LOS, was statistically supported. Therefore, according to the statistical analyses, there appeared to be no association of the presence of fungal DNA related to any of the variables. Therefore, the most important finding is that the molecular methods employed were more sensitive and faster than are culture methods, regardless of the variables considered in this research.

A relatively novel aspect of this study is that rather than target specific populations for molecular analyses with a priori knowledge of patient factors already associated with increased incidence of fungal infection [28, 29], molecular testing was performed devoid of patient information and results were then compared with patient data. The blind nature of this study demonstrated the sensitivity of molecular techniques that can be included in diagnostic settings, with less training than is necessary for specialized technicians who identify fungi based on phenotypic growth. Hence, the methods utilized in this report are potentially translatable to the clinical setting. In fact, much of the protocol can be automated, thus increasing consistency and decreasing the time and expense for each assay.

The findings of this study support the premise that molecular analyses provide sensitive and rapid detection of pathogens [2, 24, 29], including in those situations where other methods (e.g., culturing) are inadequate for the rapid detection of a diversity of organisms. The relatively inexpensive methods, open-source data investigation, and potential for high-throughput analyses of various tissue samples suggest that the methods presented in this study are prospectively translatable to a clinical setting. Additionally, identification can be accomplished in much less time (1–2 days) than with culture methods (1–2 weeks). The decreased time to diagnosis has the potential to reduce morbidity and mortality. This study provides insights into the potential number of false-negatives in culture-dependent investigations of fungal infections reported elsewhere. For example, a recent report of fungal infections within a network of 22 hospitals found that, of the 7759 fungal blood cultures performed from 2004 to 2013, 97 were positive for fungal species (1.25 %) [44]. Culturing methods were similar to those reported within this study. If 6.5 % of the culture-negative specimens contained fungi (as per the results presented here), then potentially a large number of false-negatives may occur annually. Molecular testing of the millions of culture-negative samples in the US could lead to the detection and prompt appropriate treatment of patients infected by pathogenic fungi. Thus, the results reported within this study demonstrate the potential for molecular diagnostics to enhance the sensitivity of fungal detection, and therefore, improve patient outcomes.

## Conclusions

Polymerase chain reaction amplification coupled with DNA sequencing can provide valuable diagnostic information for patients whose specimens have resulted in negative results from culturing assays. The tests provide valuable and rapid information, and have the potential to reduce morbidity and mortality.
